# Pathogenic gain-of-function mutations in the prodomain and C-terminal domain of PCSK9 inhibit LDL binding

**DOI:** 10.3389/fphys.2022.960272

**Published:** 2022-09-14

**Authors:** Samantha K. Sarkar, Angela Matyas, Ikhuosho Asikhia, Zhenkun Hu, Mia Golder, Kaitlyn Beehler, Tanja Kosenko, Thomas A. Lagace

**Affiliations:** ^1^ Department of Biochemistry, Microbiology, and Immunology, Faculty of Medicine, University of Ottawa, Ottawa, ON, Canada; ^2^ University of Ottawa Heart Institute, Ottawa, ON, Canada

**Keywords:** LDLR, low density lipoprotein receptor, LDL-C, LDL-cholesterol, PCSK9, proprotein convertase subtilisin/kexin type 9, FH, familial hypercholesterolemia, GOF, gain-of-function

## Abstract

Proprotein convertase subtilisin/kexin type-9 (PCSK9) is a secreted protein that binds and mediates endo-lysosomal degradation of low-density lipoprotein receptor (LDLR), limiting plasma clearance of cholesterol-rich LDL particles in liver. Gain-of-function (GOF) point mutations in *PCSK9* are associated with familial hypercholesterolemia (FH). Approximately 30%–40% of PCSK9 in normolipidemic human plasma is bound to LDL particles. We previously reported that an R496W GOF mutation in a region of PCSK9 known as cysteine-histidine–rich domain module 1 (CM1) prevents LDL binding *in vitro* [Sarkar et al., J. Biol. Chem. 295 (8), 2285–2298 (2020)]. Herein, we identify additional GOF mutations that inhibit LDL association, localized either within CM1 or a surface-exposed region in the PCSK9 prodomain. Notably, LDL binding was nearly abolished by a prodomain S127R GOF mutation, one of the first *PCSK9* mutations identified in FH patients. PCSK9 containing alanine or proline substitutions at amino acid position 127 were also defective for LDL binding. LDL inhibited cell surface LDLR binding and degradation induced by exogenous PCSK9-D374Y but had no effect on an S127R-D374Y double mutant form of PCSK9. These studies reveal that multiple FH-associated GOF mutations in two distinct regions of PCSK9 inhibit LDL binding, and that the Ser-127 residue in PCSK9 plays a critical role.

## Introduction

Low-density lipoprotein receptor (LDLR) binds and mediates clathrin-dependent endocytosis of several biologically important ligands, most notably apolipoprotein (apo) B100 present in circulating LDL particles. LDLR-mediated uptake in liver hepatocytes is the major conduit for plasma clearance of LDL-cholesterol (LDL-C), the primary risk factor for cardiovascular disease (Goldstein and Brown, 2009). LDLR expression is regulated at multiple levels in the maintenance of cellular and whole-body cholesterol homeostasis. One such mechanism involves secreted proprotein convertase subtilisin/kexin type-9 (PCSK9), which binds LDLR on the cell surface and promotes its degradation in lysosomes/late endosomes (Horton et al., 2009; Stein and Raal, 2013). Novel LDL-C lowering therapies targeting PCSK9 have received regulatory approval, using injectable monoclonal blocking antibodies or siRNA to inhibit PCSK9 function and raise hepatic LDLR expression (Sabatine et al., 2017; Catapano et al., 2020; Katzmann et al., 2020).

Depletion of cholesterol in cell membranes leads to activation of the transcription factor sterol regulatory element-binding protein (SREBP)-2, resulting in increased transcription of *LDLR* along with cholesterol biosynthetic genes (Horton et al., 2002; Brown and Goldstein, 2009). Paradoxically, *PCSK9* is also an SREBP-2 target gene (Horton et al., 2003; Dubuc et al., 2004), which suggests a futile cycle of LDLR production and PCSK9-mediated degradation in hepatocytes upon SREBP-2 activation. However, temporal changes in plasma LDL-C are minimal in healthy humans, while plasma PCSK9 levels fluctuate widely, following fasting-feeding and circadian patterns synchronous with hepatic cholesterol synthesis (Browning and Horton, 2010; Persson et al., 2010). Therefore, post-transcriptional regulation likely limits PCSK9’s ability to mediate LDLR degradation, yet these mechanisms remain poorly understood.

As a secreted protein, PCSK9 could be influenced by factors within the intravascular fluid. Notably, studies have shown that substantial amounts of plasma PCSK9 are bound to lipoproteins, with demonstrated avidity towards both LDL (Tavori et al., 2013a; Tavori et al., 2013b; Kosenko et al., 2013) and high-density lipoprotein particles (Burnap et al., 2021). PCSK9’s binding to LDL has been shown to involve a protein-protein interaction with apoB100, with measured affinity approximating that of the PCSK9-LDLR interaction (∼150–600 nM) (Kosenko et al., 2013; Hori et al., 2015). In addition, PCSK9 has been demonstrated to bind apoB100 in the early secretory pathway of HepG2 cells (Sun et al., 2012). Conflicting evidence has been reported regarding the effect of LDL association on PCSK9 activity. Cell culture studies support that LDL inhibits the ability of PCSK9 to bind and mediate degradation of cell surface LDLRs (Fisher et al., 2007; Kosenko et al., 2013; Galvan and Chorba, 2019); however, it has also been reported that LDL binding protects PCSK9 from inactivating furin-mediated proteolysis and induces a more potent oligomeric form (Tavori et al., 2013b; Fazio et al., 2017).

**FIGURE 1:**
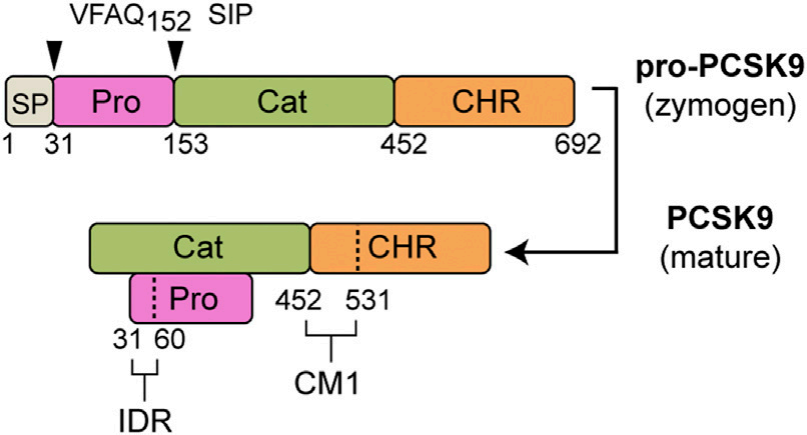
PCSK9 domain structure. Following removal of a signal peptide (SP: aa 1–30, grey) human pro-PCSK9 undergoes autocatalytic cleavage after Gln-152 resulting in mature PCSK9 consisting of a prodomain (Pro, aa 31–152, magenta), catalytic domain (Cat, aa 153–451, green) and CHR domain (aa 452–692, orange). Highlighted are two regions identified as important to LDL binding (Kosenko et al., 2013; Sarkar et al., 2020): 1) an N-terminal IDR in the prodomain (aa 31–60), and 2) CM1 within the C-terminal domain.

Mature secreted PCSK9 consists of two segments: a prodomain (∼14 kDa) and a larger segment (∼60 kDa) containing the catalytic domain and C-terminal cysteine-histidine–rich (CHR) domain (Hampton et al., 2007; Kwon et al., 2008) (Figure 1). Following autocatalytic cleavage in the endoplasmic reticulum (ER), the excised N-terminal prodomain remains non-covalently attached and obstructs substrate access to the protease active site (Cunningham et al., 2007; Seidah et al., 2013). Accordingly, PCSK9’s inherent catalytic function is not required for LDLR degradation (McNutt et al., 2007). Instead, PCSK9 acts as a binding chaperone to interfere with LDLR recycling, leading to endo-lysosomal degradation of both proteins (Zhang et al., 2007; Horton et al., 2009; Tavori et al., 2013b). Numerous *PCSK9* mutations have been identified in all three domains that confer a gain-of-function (GOF), resulting in an autosomal-dominant form of familial hypercholesterolemia (FH) (Abifadel et al., 2009; Dron and Hegele, 2017). A well-characterized example is the D374Y mutation in the PCSK9 catalytic domain that improves the primary binding interaction with the epidermal growth factor-like repeat A (EGF-A) domain of LDLR (Cunningham et al., 2007; Bottomley et al., 2008; McNutt et al., 2009). Less is known regarding functional effects of *PCSK9* GOF mutations within the prodomain and CHR domain (Dron and Hegele, 2017).

We previously identified that high-affinity LDL binding by PCSK9 requires formation of an amphipathic α-Helix within an intrinsically disordered region (IDR) in the prodomain N-terminus. Computational modeling supported that this transient helical structure aligns an intramolecular interaction with CHR domain module 1 (CM1), one of three structurally similar sub-regions of the CHR domain. In this same study, several FH-associated *PCSK9* GOF mutations localized to CM1 were found to cause defective LDL binding *in vitro* (Sarkar et al., 2020). Herein, we identify additional *PCSK9* GOF mutations that dramatically lower LDL binding, affecting residues within the same region of CM1 as well as at a second site within the prodomain. Notably, the PCSK9-LDL interaction was largely abolished by S127R, one of the first *PCSK9* mutations identified as causative for FH (Abifadel et al., 2003). Amino acid substitution analysis revealed a critical role of the native serine residue at this position in LDL binding. We discuss the potential role of defective PCSK9-LDL association in the development of hypercholesterolemia in humans.

## Materials and methods

### Reagents

We obtained fetal bovine serum (FBS) and newborn calf serum from ThermoFisher, EDTA-free Complete™ Protease Inhibitor Tablets were from Roche, Optiprep™ density gradient medium (60% w/v iodixanol) from Axis-Shield, NP-40 detergent was from Biovision and PolyJet DNA transfection reagent was from FroggaBio (Toronto, Ontario). All other reagents were from Sigma-Aldrich unless otherwise specified. Sodium mevalonate was prepared from mevalonic acid as described (Brown et al., 1978). Newborn calf lipoprotein-deficient serum (NCLPDS) (*d* > 1.215 g/ml) was prepared by ultracentrifugation (Goldstein et al., 1983). The LDLR cDNA expression vector used in these studies was pLDLR17 (Russell et al., 1989).

### Cell culture

HEK293 and Hepa-1c1c7 cells (American Type Culture Collection) were maintained in monolayer culture at 37°C and 5% CO_2_ in one of the following medium: Medium A contained DMEM (4.5 g/L glucose; Gibco) supplemented 100 U/ml penicillin and 100 μg/ml streptomycin sulfate; Medium B contained Medium A supplemented with 10% FBS (v/v); Medium C contained Medium A supplemented with 5% (v/v) NCLPDS; Medium D contained α-MEM (1.0 g/L glucose; Gibco) supplemented 100 U/ml penicillin and 100 μg/ml streptomycin sulfate; Medium E contained Medium D supplemented with 10% FBS (v/v); sterol-depleting Medium F contained Medium D supplemented with 5% (v/v) NCLPDS, 10 μM pravastatin, and 50 μM sodium mevalonate.

### Protein purification and labeling

FLAG epitope-tagged recombinant human wild-type PCSK9 along with S127R, L108R, D374Y and S127R-D374Y variants were produced in stably-transfected HEK293S cells and purified from conditioned culture medium as previously described (Kosenko et al., 2013). Fluorescently labeled wild-type PCSK9 was prepared using the DyLight800 Antibody Labeling Kit (Pierce) as per manufacturer’s instructions followed by gel filtration chromatography on a Superdex 200 10/300 GL column (GE Healthcare) to remove unbound dye.

### Antibodies

The following antibodies were used for Western blotting: A mouse hybridoma clone expressing monoclonal antibody 15A6 recognizing an epitope in the CHR domain of PCSK9 was a generous gift from J. Horton (UTSouthwestern Medical Center, Dallas, TX). The IgG fraction was purified from hybridoma culture medium by Protein A chromatography on a Profinia™ protein purification system (Bio-Rad) as per manufacturer’s instructions. Rabbit anti-serum 3143 against the C-terminal 14 amino acids of LDLR was the kind gift of J. Herz (UTSouthwestern Medical Center). Anti-actin mouse monoclonal ascites fluid (clone AC-40) was from Sigma. Mouse anti-human transferrin receptor antibody was from Life Technologies. Secondary IRDye-labeled goat anti-mouse and anti-rabbit IgG antibodies were from LI-COR Biosciences. Rabbit anti-serum 1697 raised against full-length human PCSK9 was used for immunoprecipitation and was custom produced by Biomatik (Cambridge, Ontario, Canada)

### Cultured cell harvest and Western blotting

Following treatments, cells were washed in ice-cold PBS-CM (PBS with 1 mM MgCl_2_ and 0.1 mM CaCl_2_) and whole cell extracts were prepared in Tris Lysis Buffer (50 mm Tris-Cl, pH 7.4, 150 mm NaCl, 1% NP-40, 0.5% sodium deoxycholate, 5 mm EDTA, 5 mm EGTA, 1X Complete™ protease inhibitor cocktail, 1 mM phenylmethylsulfonyl fluoride). Proteins in cell extracts or immunoprecipitates were prepared in 1X Laemmli sample buffer (Bio-Rad) supplemented with 50 mM dithiothreitol, heated for 5 min at 96°C and loaded for electrophoresis onto 8% SDS-polyacrylamide gels or 4%–12% Tris/HEPES-SDS Bolt™ precast gels (Thermo-Invitrogen). Size-separated proteins were transferred to nitrocellulose membranes (Bio-Rad). Primary antibodies (described above) and IRdye800-conjugated secondary antibodies were used to detect target proteins using the LI-COR Odyssey infrared imaging system (LI-COR Biosciences). Quantification of the intensity of the bands was obtained using Odyssey 2.0 software (LI-COR Biosciences).

### Site-directed mutagenesis

The pcDNA3-PCSK9-FLAG vector (Park et al., 2004) codes for full-length human wild type PCSK9 with a FLAG-tag epitope attached at the C-terminus and was used as the template to generate PCSK9 mutants. Mutagenesis was carried out using the QuikChange site-directed mutagenesis protocol from Stratagene (La Jolla, CA). PCR primers were custom-synthesized by Invitrogen-Life Technologies or Integrated DNA Technologies and are listed in Supplementary Table S1. All desired mutations and absence of extraneous mutations were confirmed by sequencing the entire coding region.

### Preparation of conditioned media

HEK293 cells cultured in Medium B to ∼70% confluency in 100-mm Petri dishes were transfected with a total of 3 μg of PCSK9 cDNA expression vectors using PolyJet DNA transfection reagent as per manufacturer’s instructions. At 18 h post-transfection, the cells were washed with PBS and incubated with serum-free Medium A containing 1X ITS (insulin-transferrin-selenium) cell culture supplement (Invitrogen). Conditioned media were recovered 48 h post-transfection, concentrated ∼10-fold on an Amicon Ultra-4 centrifugal filter unit with a 10-kDa membrane cutoff (Millipore) and supplemented to 25 mM Hepes pH 7.4. PCSK9 levels in conditioned media preparations were quantified by Western blot analysis using purified PCSK9 as a standard.

### LDL isolation

All procedures using human subjects received regulatory approval from the Human Research Ethics board at the University of Ottawa Heart Institute and followed Declaration of Helsinki principles. Blood samples were drawn from fasted healthy volunteers into evacuated tubes containing EDTA and plasma was separated by low-speed centrifugation. Protease inhibitors (1 mM PMSF, 50 U/ml aprotinin and Complete™ protease inhibitor cocktail) and antioxidant (20 μM butylated hydroxytoluene) were added to the cleared plasma. LDL (*d* = 1.019–1.065 g/ml) was isolated using sequential potassium bromide flotation ultracentrifugation (Havel et al., 1955) followed by extensive dialysis against phosphate-buffered saline (PBS) containing 0.25 mM EDTA. LDL isolated in this manner did not contain detectable levels of endogenous PCSK9 (data not shown), likely due to stripping of peripherally associated PCSK9 under high-salt and high *g* forces. LDL was stored at 4°C protected from light and used within 1 month. Alternatively, LDL was stored at −80°C in 10% (w/v) sucrose as described (Rumsey et al., 1992), which did not affect PCSK9 binding.

### PCSK9-LDL binding assays

Binding reactions (1.0 ml volume) each containing 500 μg LDL and approximately 1 µg PCSK9 (in concentrated conditioned media) and 0.5% BSA (w/v) in HBS-C buffer (25 mM HEPES-KOH, pH 7.4, 150 mM NaCl, 2 mM CaCl_2_) were incubated at 37°C for 1 h. LDL-bound and free PCSK9 were then separated by Optiprep gradient ultracentrifugation as previously described (Sarkar et al., 2020). PCSK9 present in the collected LDL-containing fraction was immunoprecipitated with rabbit anti-serum 1697 directed against human PCSK9 prior to Western blot analysis. To determine relative LDL binding affinity of PCSK9 mutants, competition binding curves were generated as previously described (Sarkar et al., 2020). The amount of competitor PCSK9 protein required for 50% inhibition of fluorophore-labeled PCSK9 binding (K*i*) to LDL was determined by fitting data to a sigmoidal dose response curve using nonlinear regression (GraphPad Prism 9 software).

### Cellular LDLR binding and degradation

For cell surface LDLR binding assays, HEK293 cells were cultured in Medium B in 6-well dishes to 70% confluency, then transiently transfected with 1 μg wild-type human LDLR plasmid per well using PolyJet DNA transfection reagent as per manufacturer’s instructions. After 18–20 h, cell medium was replaced with lipoprotein-deficient Medium C containing purified PCSK9-D374Y or S127R-D374Y double mutant (0.5 μg/ml) preincubated with LDL (1 mg/ml) for 1 h at 37°C. Dishes were incubated for a further 2 h in the presence of 50 μM chloroquine prior to cell harvest and Western blot analysis. To assess LDLR degradation, Hepa1c1c7 cells in 60 mm dishes were cultured in Medium E to 60% confluency, then switched to sterol-depleting Medium F for 18–20 h. Recombinant PCSK9, either in conditioned medium or in purified form, were added to the medium and cells incubated at 37°C for 4 h. Whole cell extracts were prepared for Western blotting as above. For some assays, cell surface proteins were biotinylated with Sulfo-NHS-SS-Biotin (Campbell Science, Rockford, Illinois, United States), and captured from whole cell extracts using Neutravidin agarose beads (Pierce) as previously described (Nguyen et al., 2014).

### Statistics

Data are presented as the mean ± SEM. Two-sided statistical analysis was determined by Student’s *t*-test using GraphPad Prism 9 software. Data used measurements from at least two distinct sample preparations for each component.

## Results

### Identification of GOF mutations in the PCSK9 prodomain that inhibit LDL binding

**FIGURE 2:**
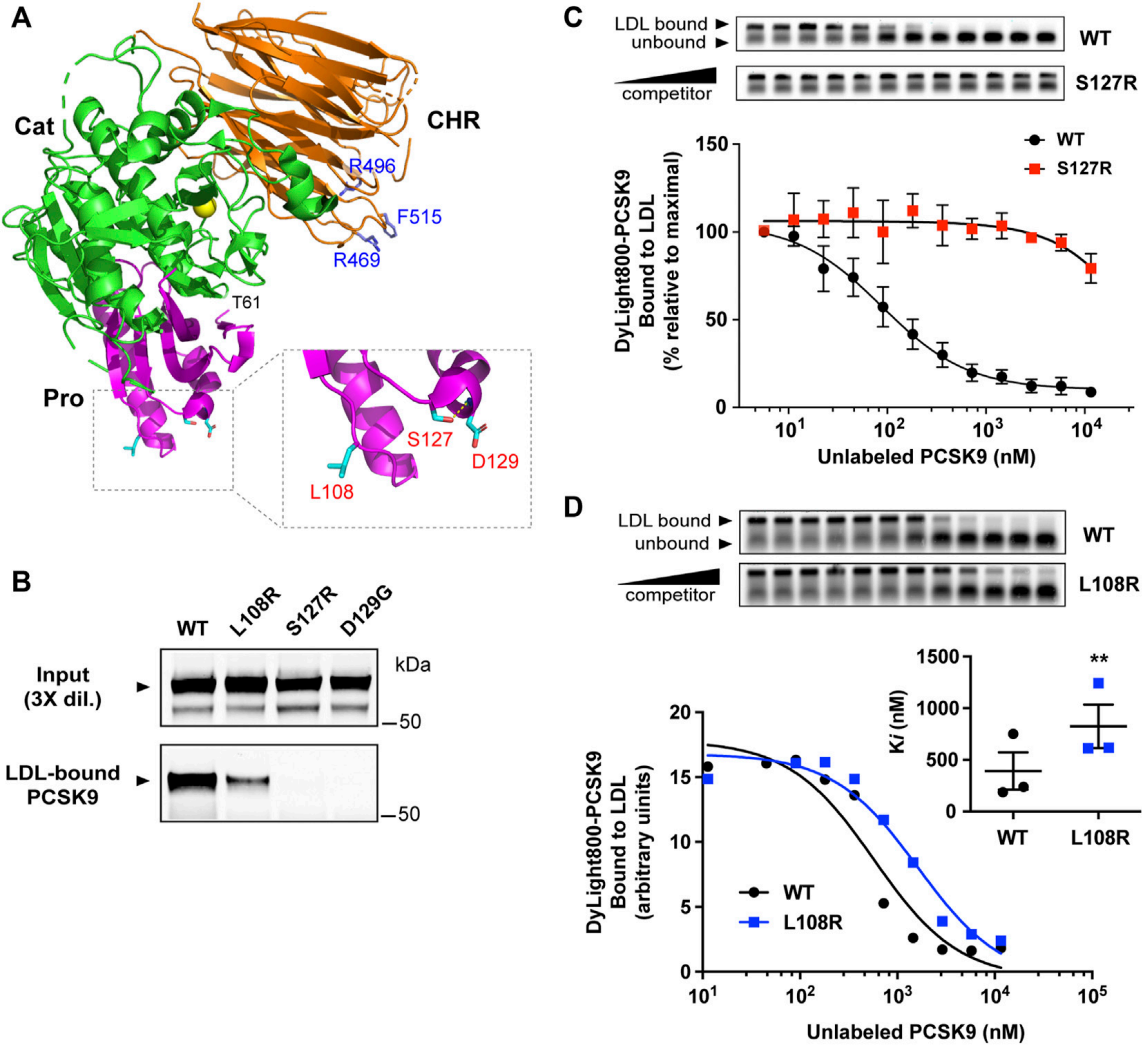
GOF mutations in the PCSK9 prodomain inhibit LDL binding function. **(A)** Crystal structure of PCSK9 (PDB ID: 2QTW) (Hampton et al., 2007) generated with PyMOL (http://www.pymol.org). Color scheme: Prodomain (Pro, magenta), catalytic domain (Cat, green), CHR domain (orange). Highlighted in *blue* are residues in the CHR domain affected by *PCSK9* GOF mutations known to inhibit LDL binding (Sarkar et al., 2020). Box: details of prodomain region showing close proximity of residues associated with FH. Side-chain sticks of L108, S127, and D129 are highlighted (cyan). Also depicted is a hydrogen bond involving the side-chain of S127 and main-chain amino group of D129 (yellow broken line). **(B)** Conditioned cell culture medium containing 1.0 μg/ml wild-type PCSK9 (WT) or indicated mutants were incubated with PCSK9-free human LDL (0.5 mg/ml) prior to density gradient-ultracentrifugation and immunoprecipitation and Western blot analysis of LDL-containing fractions. Shown is a representative experiment (*n* = 3) **(C)** Competition binding of WT-PCSK9 and S127R mutant PCSK9 to LDL. Dylight800-labeled WT-PCSK9 was incubated with LDL in the presence of increasing concentrations of unlabeled PCSK9. Reaction mixtures were separated on agarose gels (*top*) and fluorophore-labeled PCSK9 binding to LDL was quantified and fitted to competition binding curves using non-linear regression (*n* = 3) (*bottom*). **(D)** Competition binding of WT-PCSK9 and L108R mutant PCSK9 to LDL. Analysis was carried out as in **(C)** and shown is a representative experiment. Inhibitor constants (Ki) obtained from curves are graphically represented (*inset*). Error bars represent SEM (*n* = 3). Significant change in LDL binding of L108R mutant PCSK9 compared with WT-PCSK9 was determined by Student’s *t*-test: **, *p* < 0.01.

Figure 1 shows the domain structure of PCSK9, highlighting regions previously shown to contain elements critical to LDL binding, namely an N-terminal IDR in the prodomain and CM1 in the CHR domain (Kosenko et al., 2013; Sarkar et al., 2020). We previously identified several GOF mutations in CM1 that confer lowered (R469W and F515L) or abolished (R496W) LDL binding ability *in vitro* (Sarkar et al., 2020). Although distant from these residues, several GOF mutations in the prodomain (L108R, S127R, and D129G) are localized on approximately the same molecular plane (Figure 2A). To assess LDL association, plasmid constructs expressing either wild-type (WT) PCSK9 or prodomain mutant versions were transiently transfected into HEK293 cells. Concentrated conditioned medium containing secreted PCSK9 (1 μg/ml) was incubated with PCSK9-free human LDL (0.5 mg/ml) followed by iodixonal density gradient centrifugation to separate LDL-bound and unbound PCSK9. Immunoprecipitation and Western blot analysis of the LDL-containing fractions showed that all three mutant PCSK9 proteins had diminished ability to bind LDL (Figure 2B). This effect was greatest for the S127R and D129G mutations, which appeared to nearly abolish LDL binding. Competition binding experiments showed that the S127R mutation lowered LDL binding affinity by at least two orders of magnitude (Figure 2C), whereas the L108R mutation in PCSK9 decreased LDL binding affinity by ∼2.5-fold (Figure 2D).

### The Ser-127 residue in PCSK9 is critical to its LDL binding function

**FIGURE 3:**
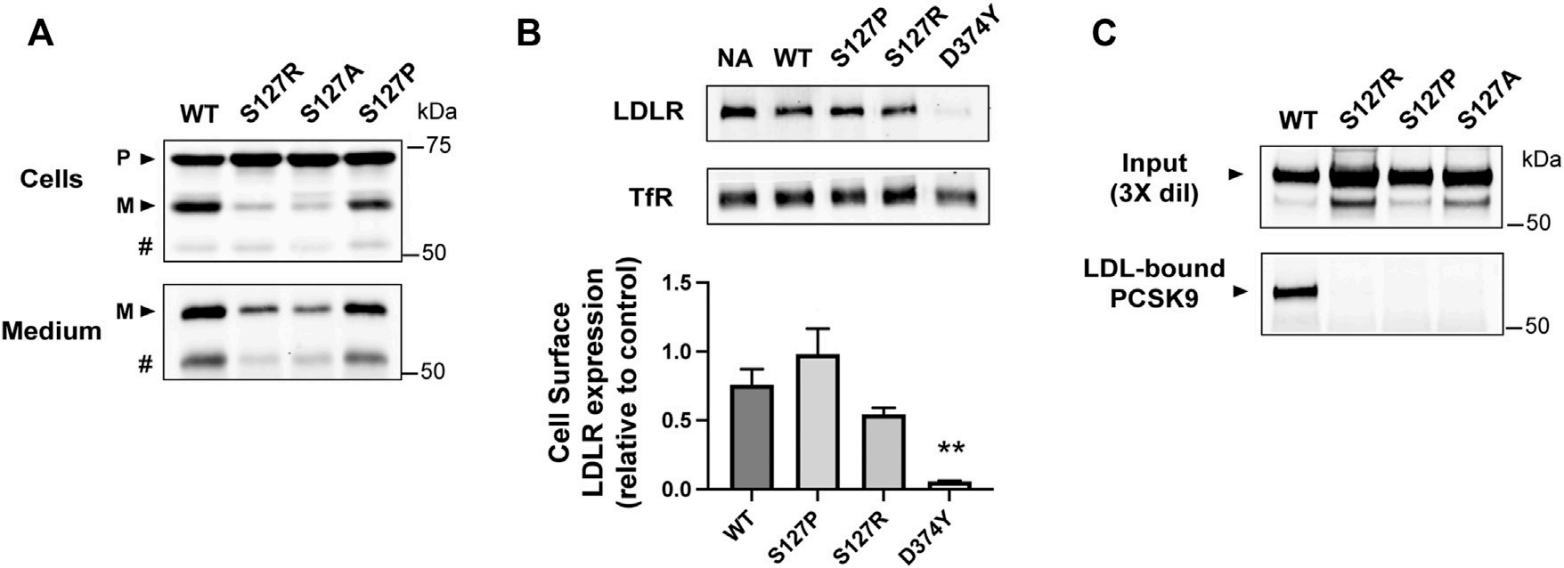
Ser-127 residue is critical to LDL binding function of PCSK9. **(A)** 24 h post-transfection, HEK293 cells transiently expressing WT-PCSK9 or indicated S127 mutants were harvested and cell lysates and medium immunoprecipitates were analyzed by 8% SDS-PAGE and western blotting to detect precursor (P) and mature (M) forms of PCSK9. # indicates a faster-migrating form of PCSK9 consistent with furin-mediated proteolysis. Shown is a representative experiment (*n* = 3) **(B)** Mouse Hepa-1c1c7 cells cultured in sterol-depleting medium were treated for 4 h with WT or indicated mutant forms of PCSK9 (2.5 μg/ml). Biotinylated cell surface LDLRs were isolated and quantified by Western blotting using transferrin receptor (TfR) as a loading control. Shown are representative western blots (*top*) with densitometric analysis of three independent experiments (*bottom*). Error bars represent SEM (*n* = 3). Significant change in LDLR expression compared to WT-PCSK9 was determined by Student’s *t*-test: **p < 0.01. **(C)** Conditioned cell culture medium containing WT-PCSK9 (WT) or indicated mutants were incubated with LDL prior to density gradient-ultracentrifugation and immunoprecipitation and Western blot analysis of LDL-containing fractions. Shown is a representative experiment (*n* = 3)

Known functional effects of the S127R mutation include a delay in cellular PCSK9 auto-processing and secretion (Benjannet et al., 2004) and increased LDLR binding/degradation (Cunningham et al., 2007; Pandit et al., 2008). Previous studies have shown that these effects can be normalized by substitutions of proline (S127P) or alanine (S127A), respectively (Benjannet et al., 2004; Pandit et al., 2008). As expected, S127R and S127A PCSK9 had slower auto-processing and secretion compared to WT PCSK9 when transiently expressed in HEK293 cells, whereas proline substitution (S127P) reversed this effect (Figure 3A). To assess cell surface LDLR degradation, mouse Hepa-1c1c7 hepatoma cells were treated for 4 h with PCSK9 present in conditioned medium (2.5 μg/ml), followed by cell surface biotinylation and immunoblot analysis. The S127R mutation modestly increased PCSK9-mediated LDLR degradation and far less potently than D374Y, in agreement with previous studies (Pandit et al., 2008), whereas LDLR degradative activity of PCSK9-S127P was comparable to that of WT-PCSK9 (Figure 3B). In terms of LDL binding, all three Ser-127 PCSK9 variants tested (S127R, S127A, and S127P) were equally defective, with no detectable PCSK9 protein in an LDL-containing fraction compared to WT PCSK9 control (Figure 3C).

### The S127R mutation renders PCSK9 resistant to inhibition by LDL

**FIGURE 4:**
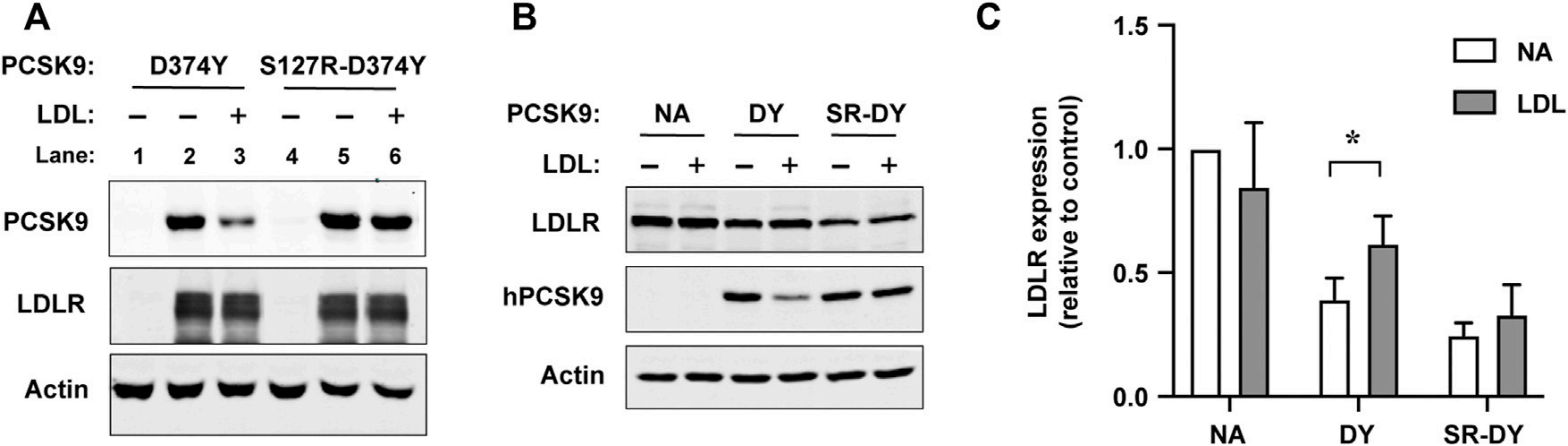
LDL does not inhibit LDLR binding and degradation mediated by PCSK9 harboring the S127R mutation. **(A)** Cell surface LDLR binding/uptake of PCSK9. HEK293 cells transiently transfected with vector control or LDLR were incubated for 2 h in lipoprotein-deficient medium containing 0.5 μg/ml of the indicated mutant PCSK9 preincubated in the absence (−) or presence (+) of LDL (1.0 mg/ml). Cell lysates were analyzed by SDS-PAGE and Western blotting as described in methods. Shown is a representative experiment (*n* = 3) **(B)** LDLR degradation induced by exogenous PCSK9. Mouse Hepa-1c1c7 cells cultured in sterol-depleting medium were treated for 4 h with either D374Y (DY) or S127R-D374Y (SR-DY) double mutant PCSK9 (0.5 μg/ml) preincubated in the absence (−) or presence of LDL (1 mg/ml). Cell lysates were analyzed by SDS-PAGE and western blotting as described in methods. **(C)** Densitometric analyses of Western blot in **(B)** *.* Error bars represent SEM (*n* = 3). Significant change in LDLR expression compared to no addition (NA) control was determined by Student’s *t*-test: *p < 0.05.

We next asked whether introduction of the S127R mutation prevented an ability of LDL to inhibit the binding of PCSK9 to cell surface LDLRs. For these experiments we employed PCSK9-D374Y since this GOF mutation does not affect LDL binding and the increased affinity to LDLR overcomes detection limitations in Western blot analysis, allowing for physiological concentrations of PCSK9 (500 ng/ml) and LDL (1 mg/ml). LDLR-dependent uptake of PCSK9-D374Y or an S127R-D374Y double mutant form was assessed in HEK293 cells transiently overexpressing recombinant LDLR. PCSK9 proteins were first preincubated with or without LDL and then added to cells for 2 h in the presence of chloroquine to prevent endo-lysosomal degradation of internalized PCSK9. Due to low endogenous PCSK9 and LDLR expression, minimal PCSK9 was detectable by immunoblot in lysates of non-transfected HEK293 cells (Figure 4A, lanes 1 and 4). In cells overexpressing LDLR, uptake of both PCSK9 proteins was robust and equivalent (Figure 4A, lanes 2 and 5). In the presence of LDL, LDLR-dependent uptake of PCSK9-D374Y was substantially inhibited (Figure 4A, lane 3), whereas that of the S127R-D374Y double mutant PCSK9 was unaffected (Figure 4A, lane 6). Similarly, when added to the culture medium of Hepa-1c1c7 cells, LDL significantly inhibited the ability of PCSK9-D374Y to induce LDLR degradation but had no effect on the S127R-D374Y double mutant PCSK9 (Figures 4B,C).

### Multiple FH-associated mutations in the CM1 region of PCSK9 inhibit LDL binding

**FIGURE 5:**
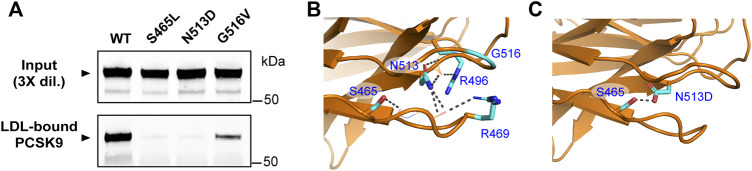
Multiple GOF mutations in the CM1 region of PCSK9 inhibit LDL binding. **(A)** Conditioned cell culture medium containing 1.0 μg/ml wild-type PCSK9 (WT) or indicated mutants were incubated with LDL (0.5 mg/ml) prior to density gradient-ultracentrifugation and immunoprecipitation and Western blot analysis of LDL-containing fractions. Shown is a representative experiment (*n* = 3) **(B)** Details of CM1 region showing close proximity of residues affected by FH mutations. The side-chains of S465, R469, R496, and N513 are positioned to form main-chain hydrogen bonds (grey broken lines). **(C)** Point mutation of Asn-513 to Asp was made *in silico* using the Mutagenesis Wizard module of PyMOL (http://www.pymol.org) with rotamer (orientation) of highest frequency. In the model, Asp-513 is positioned to form a hydrogen bond with Ser-465 (grey broken line).

As mentioned above, the prodomain IDR in PCSK9 has been identified to play an important structural role in LDL binding while several GOF mutations (R469W, R496W, and F515L) in the CM1 region of the CTD were found to inhibit LDL association *in vitro* (Sarkar et al., 2020). Numerous additional *PCSK9* mutations associated with FH have been identified within these regions (Abifadel et al., 2009; Dron and Hegele, 2017). We generated corresponding PCSK9-encoding plasmids for transient overexpression in HEK293 cells and collected the conditioned medium to assess LDL binding of these PCSK9 variants. As summarized in Table 1, negligible effects on LDL association compared to WT control were seen for selected mutations located in the IDR and other regions of the prodomain. However, several GOF mutations affecting residues within CM1 were found to dramatically lower PCSK9’s ability to bind LDL. Specifically, S465L and N513D mutations in PCSK9 nearly abolished LDL binding, whereas a G516V mutation decreased LDL binding by ∼70% (Figure 5A). The highest-resolution X-ray crystal structure of PCSK9 (Hampton et al., 2007) shows that the side-chains of affected residues Ser-465, Arg-469, Arg-496, and Asn-513 are each positioned to form hydrogen bond interactions with the protein backbone in two adjacent loop regions in CM1 (Figure 5B). Interestingly, Asn-513 modeled as aspartate (as in the N513D mutation) is positioned to form a hydrogen bond with nearby Ser-465 (Figure 5C). Therefore, the lack of LDL binding for the N513D GOF mutation could involve changes in the intramolecular interactions of Ser-465.

**TABLE 1:** Effect on LDL binding *in vitro* of PD- and CM1-localized *PCSK9* mutations associated with total cholesterol and LDL-C. All mutations are associated with hypercholesterolemia and designated as GOF unless indicated. Loss-of-function (LOF) mutations are associated with hypocholesterolemia. Selected mutations are described in references herein and/or listed in the NCBI ClinVar database (https://www.ncbi.nlm.nih.gov/clinvar/?term=PCSK9%5Bgene%5D&redir=gene).

*PCSK9* mutations in PD and CM1-LDL binding		
Decreased >90%	Decreased 50%–90%	Unchanged
*PD*	*PD*	*PD*
S127R	L108R	E32K
D129G		D35Y
	*CM1*	R46L (LOF)
*CM1*	R469W	E48K
S465L	F515L	A53V (LOF)
R496W	G516V	E54A
N513D		E57K (LOF)
		G59R
		R96L
		R96C
		*CM1*
		P467A
		I474V (LOF)
		R499H
		A514T

## Discussion

Substantial amounts of plasma PCSK9 are bound to lipoproteins, including ∼30%–40% that is bound to LDL particles (Tavori et al., 2013b; Kosenko et al., 2013); however, the molecular detail and functional significance of the PCSK9-LDL interaction has yet to emerge. Herein, we demonstrate that numerous *PCSK9* GOF mutations prevent or severely inhibit LDL binding *in vitro*. The residues affected by these mutations cluster at two surface-exposed sites in the prodomain (Figure 2A) and CM1 (Figure 5B), thus pinpointing protein regions critical to the PCSK9-LDL interaction. Furthermore, since the identified LDL-binding–defective *PCSK9* mutations are associated with FH, these data lend support to the notion that LDL association exerts an inhibitory effect on the ability of PCSK9 to mediate hepatic LDLR degradation in humans.

### Potential mechanism of PCSK9 inhibition by LDL

Several studies have shown that LDL inhibits PCSK9’s ability to bind and mediate LDLR degradation in cultured cells (Fisher et al., 2007; Kosenko et al., 2013; Galvan and Chorba, 2019). While the mechanism remains unknown, current evidence supports that LDL does not exert a direct blocking effect on the PCSK9-LDLR interaction. Specifically, we previously demonstrated that PCSK9 can bind to the LDLR EGF-A domain and LDL simultaneously, and that LDL maintains its ability to inhibit binding of PCSK9 to mutant LDLRs that cannot bind LDL (Kosenko et al., 2013). In the current study, we report that a single point mutation in PCSK9 (S127R) prevented LDL from inhibiting PCSK9-mediated LDLR binding and degradation in cultured cells (Figure 4). A second possibility is that LDL binding stabilizes a cryptic autoinhibited conformation involving the prodomain IDR (aa 31–60; Figure 1) (Benjannet et al., 2010; Holla et al., 2011; Seidah, 2019). Previous work showed that deletion of the N-terminal 21 amino acids in the IDR increased PCSK9-LDLR binding affinity by >7-fold while abolishing PCSK9’s ability to bind LDL (Kwon et al., 2008; Kosenko et al., 2013). This stretch of amino acids contains 12 acidic residues (Asp or Glu) along with a tyrosine residue that can be post-translationally modified by O-sulfation, adding to the negative charge. Similar acidic IDRs have well-established roles in regulating protein-protein interactions in processes such as cell signaling and gene transcription (Sigler, 1988; Wright and Dyson, 2015), often modulated by post-translational modifications or structural shifts within these domains (Lee et al., 2000; Borcherds et al., 2014; Krois et al., 2016). Recently, this mode of regulation has been implicated in lipoprotein metabolism. Structure/function studies showed that an acidic N-terminal IDR in glycosylphosphatidylinositol-anchored high-density lipoprotein–binding protein 1 (GPIHBP1) contacts a basic patch in lipoprotein lipase (LPL) *via* electrostatic steering, with a sulfotyrosine residue in GPIHBP1’s IDR further stabilizing the protein-protein interaction (Kristensen et al., 2018). This in turn prevents LPL inactivation by angiopoietin-like protein 4, thereby increasing lipolysis of triglyceride-rich lipoproteins (Kristensen et al., 2021). In the case of PCSK9, it was recently shown that the N-terminal acidic IDR can adopt transient α-helical structure (Ultsch et al., 2019; Sarkar et al., 2020). Computational modeling supported this structural shift aligns an intramolecular interaction with CM1, which is enriched in basic residues (Sarkar et al., 2020). Introduction of a helix-breaking proline residue in this region did not affect PCSK9’s interaction with LDLR; however, LDL binding affinity was decreased by >4-fold (Ultsch et al., 2019; Sarkar et al., 2020). Hence, it is tempting to speculate that the N-terminal acidic IDR contacts CM1 in a transient manner via electrostatic steering, resulting in allosteric autoinhibition of PCSK9-LDLR binding. A coil-to-helix structural shift together with LDL binding could then stabilize this autoinhibited conformational state in PCSK9.

### GOF mutations reveal regions in PCSK9 critical to LDL binding

If LDL binding inhibits PCSK9 *in vivo*, then *PCSK9* mutations that disable LDL binding would result in hyperactive GOF variants. Along with a previous study (Sarkar et al., 2020), we have now characterized a total of six LDL-binding–defective *PCSK9* mutations (S465L, R469W, R496W, N513D, F515L, and G516V) identified in FH patients and localized to the CM1 region of the CHR domain. Notably, the side-chains of Ser-465, Arg-469, Arg-496, and Asn-513 are each positioned to form hydrogen bonds to the protein backbone within two adjacent loop regions (Figure 5B) (Hampton et al., 2007), suggesting that GOF mutations affecting these residues alter local structural arrangements critical to LDL association. We also identified additional LDL-binding–defective *PCSK9* GOF mutations involving closely juxtaposed residues in the prodomain (L108R, S127R, and D129G). Although distant from the affected residues in CM1, this second site is localized on approximately the same molecular plane (Figure 2A). Therefore, the LDL binding interface in PCSK9 could encompass multiple domains and exert a global conformational effect on the LDLR binding interface in the catalytic domain. It is currently unknown if any of the identified LDL-binding–defective *PCSK9* GOF mutations affect residues making direct contacts with apoB100. It is also possible that some of these residues are involved in membrane lipid interactions with the LDL particle, although a lipid-binding function of PCSK9 has yet to be established.

### The S127R mutation in *PCSK9* and its functional effects in FH

The *PCSK9* S127R mutation was originally identified in a French family with severely elevated LDL-C and an absence of genetic abnormalities in *LDLR* or *APOB*, the only genes known at the time to be associated with autosomal-dominant hypercholesterolemia (Abifadel et al., 2003). This landmark discovery provided the initial link between FH and PCSK9, a newly discovered member of the proprotein convertase family of serine proteases (Seidah et al., 2003). Subsequent studies of the S127R mutation have characterized multi-faceted functional effects in PCSK9, possibly resulting in additive or synergistic effects on plasma LDL-C levels. *In vivo* kinetics studies carried out on two French subjects carrying the S127R mutation showed increased production of apoB100-containing lipoproteins, hinting at a direct role of the mutant PCSK9 in promoting apoB secretion in liver (Ouguerram et al., 2004). In the current study we provide evidence that PCSK9-S127R is severely defective in its ability to bind apoB100 in LDL particles (Figure 2B), suggesting that increased apoB100 production observed in S127R patients involves indirect mechanisms. Decreased LDLR activity could be a contributing factor since increased hepatic apoB production has also been observed in FH patients with *LDLR* mutations (Cummings et al., 1995; Tremblay et al., 2004; Millar et al., 2005). While there is evidence that the S127R mutation enhances PCSK9’s ability to bind LDLR and induce LDLR degradation in cells, this effect is modest compared to the D374Y mutation (Figure 3B) (Cunningham et al., 2007; Pandit et al., 2008; Martin et al., 2020) and thus unlikely to account for severe hypercholesterolemia in S127R patients. Molecular modeling supports that Arg-127 forms secondary contacts with the β-Propeller domain in LDLR leading to improved LDLR binding/degradation in the endosomal compartment (Lo Surdo et al., 2011). In agreement, alternate Ser-127 substitutions did not increase cellular PCSK9-mediated LDLR degradation (Pandit et al., 2008). Introduction of positive-charged Arg-127 also improved PCSK9 binding to heparin sulfate proteoglycans (Galvan and Chorba, 2019), which could improve LDLR interactions at the surface of hepatocytes (Gustafsen et al., 2017). By contrast, we present evidence that loss of the native serine residue underlies the negative effect of the S127R mutation on PCSK9-LDL binding (Figure 3C). Due to its small size, serine is relatively common in tight turns, where the side-chain hydroxyl can form a hydrogen bond with the protein backbone, in effect mimicking proline (Betts et al., 2003). Indeed, a high-resolution X-ray crystal structure of PCSK9 shows that Ser-127 is located within a tight turn and is positioned to form a hydrogen bond with the backbone amine group of neighboring Arg-129 (Figure 2B) (Hampton et al., 2007). While an S127P substitution allowed for normal PCSK9 auto-processing and secretion (Benjannet et al., 2004) (Figure 3A), it did not rescue defective LDL binding (Figure 3C). Therefore, the PCSK9-LDL interaction may require more precise structural features or additional contacts involving Ser-127.

### Potential role of defective PCSK9-LDL binding in FH pathogenesis

Plasma levels of LDL reflect a balance between rates of clearance and production (Brown and Goldstein, 1981). In healthy humans, a majority of LDL is produced through intravascular lipolysis and remodeling of very low-density lipoprotein (VLDL), a large triglyceride-rich apoB100-containing lipoprotein secreted from liver (Borén et al., 2022). In addition to controlling plasma LDL levels, hepatic LDLR contributes to the clearance of VLDL remnant particles that have acquired the LDLR ligand apoE (Veniant et al., 1998; Mahley and Ji, 1999; Foley et al., 2013). VLDL remnants that remain in circulation undergo further lipolysis and remodeling to the short-lived intermediate density lipoprotein (IDL) and finally to LDL (Kita et al., 1982; Dallinga-Thie et al., 2010). We have previously shown that PCSK9 does not bind to VLDL in human plasma or *in vitro* (Kosenko et al., 2013), suggesting that the conversion to LDL reveals the binding site for PCSK9, as is also the case for LDLR recognition of apoB100-containing lipoprotein particles (Milne et al., 1989). Plasma PCSK9 levels in humans are decreased with fasting and increased postprandially and display a diurnal rhythm that mirror plasma levels of lathosterol (Browning and Horton, 2010; Persson et al., 2010), a marker of hepatic cholesterol synthesis and VLDL secretion (Riches et al., 1997). Thus, concurrent secretion of PCSK9 and VLDL could serve to increase plasma excursion of VLDL remnants by limiting hepatic clearance *via* LDLR. Among various lipoprotein classes, plasma PCSK9 level in human subjects was found to be most highly positively correlated with IDL (Kwakernaak et al., 2014). Furthermore, *in vivo* kinetic studies showed that PCSK9 inhibition with monoclonal blocking antibodies decreased the production rates of IDL and LDL, suggestive of improved clearance of VLDL remnants (Reyes-Soffer et al., 2017; Watts et al., 2017). These studies support that PCSK9 activity promotes VLDL-to-LDL conversion in humans, which could in turn exert negative feedback control on PCSK9 through direct LDL binding. PCSK9 GOF variants that are highly defective for LDL binding would not be subject to this inhibitory mechanism, contributing to the FH phenotype. Conversely, none of the *PCSK9* LOF mutations associated with hypocholesterolemia that we have tested to date displayed enhanced LDL binding (Table 1). It should be noted, however, that structural studies showed dramatically increased coil-to-helix transition in N-terminal PCSK9-derived peptides containing R46L (Sarkar et al., 2020), an atheroprotective *PCSK9* LOF mutation found in ∼3% of Caucasians (Cohen et al., 2006; Verbeek et al., 2017). As mentioned above, evidence supports this structural shift in the prodomain IDR improves PCSK9-LDL binding affinity (Sarkar et al., 2020).

## Conclusion

Assessment of LDL’s effect on PCSK9 activity *in vivo* is complex since increased LDL-C uptake suppresses hepatic LDLR and PCSK9 expressions *via* negative feedback on SREBP-2 activity (Brown and Goldstein, 2009; Horton et al., 2009). In this regard, GOF PCSK9 mutations selectively defective for LDL binding could provide useful molecular tools to assess direct versus indirect effects of elevated LDL on PCSK9 plasma levels and activity. We previously showed that the R496W mutation in PCSK9 does not affect LDLR binding/degradative function (Sarkar et al., 2020), and preliminary analysis indicates this is also the case for the N513D mutation (*Z. Hu, unpublished data*). A previous study analyzed the S465L *PCSK9* mutation identified in an FH family and found no discernable effect on LDLR function in cultured peripheral blood mononuclear cells, leading the study authors to conclude that the novel mutation was likely not pathogenic (Ruotolo et al., 2014). The results of the current study support that PCSK9-LDL binding analysis should also be included for assessment of potential FH-associated gene mutations in *PCSK9* and *APOB* that do not affect the respective LDLR interactions.

## Data Availability

The original contributions presented in the study are included in the article/Supplementary Material, further inquiries can be directed to the corresponding author.

## Abbreviations

aa, amino acids; Apo, apolipoprotein; CHR, cysteine-histidine rich; CM1, CHR domain module 1; EGF-A, epidermal growth factor-like repeat A; ER, endoplasmic reticulum; FH, familial hypercholesterolemia; GOF, gain-of-function; IDR, intrinsically disordered region; LDL-C, LDL-cholesterol; LDLR, low density lipoprotein receptor; LOF, loss-of-function; PCSK9, proprotein convertase subtilisin/kexin type 9; PDB, protein data bank; SREBP, sterol regulatory element-binding protein; VLDL, very low-density lipoprotein.
