# Supplementary material for: Pathogenic gain-of-function mutations in the prodomain and C-terminal domain of PCSK9 inhibit LDL binding
Source: Front Physiol. 2022 Sep 14;13:960272. doi: 10.3389/fphys.2022.960272 (PMC9515655; doi:10.3389/fphys.2022.960272)
Supplement: Supplementary file 1 [file Table1.DOCX]

**Supplemental Table 1:** Sequences of forward (F) and reverse (R) primers used to mutate C-terminally FLAG-tagged wild-type human PCSK9

E32K F: CGTGCGCAGAAGGACGAGGACG

R: CGTCCTCGTCCTTCTGCGCACG

D35Y F: GAGGACGAGTACGGCGACTACG

R: CGTAGTCGCCGTACTCGTCCTC

E48K F: TTGCGTTCCAAGGAGGACGGC

R: GGCCGTCCTCCTTGGAACGCAAGGC

E57K F: CCGAAGCACCCAAGCACGGAACCAC

R: GTGGTTCCGTGCTTGGGTGCTTCGG

G59R F: CACCCGAGCACCGAACCACAGCCAC

R: GTGGCTGTGGTTCGGTGCTCGGGTG

R96L F: GCGCACTGCCCTCCGCCTGCAGGCC

R: GGCCTGCAGGCGGAGGGCAGTGCGC

R96C F: GCGCACTGCCTGCCGCCTGCAG

R: TGCAGGCGGCAGGCAGTGC

L108R F: CCGCCGGGGATACCGCACCAAGATCCTGC

R: GCAGGATCTTGGTGCGGTATCCCCGGCGG

S127R F: GGTGAAGATGAGAGGCGACCTGCTG

R: CAGCAGGTCGCCTCTCATCTTCACC

S127A F: CCTGGTGAAGATGGCTGGCGACCTGCTG

R: CAGCAGGTCGCCAGCCATCTTCACCAGG

S127P F: CCTGGTGAAGATGCCTGGCGACCTGCTG

R: CAGCAGGTCGCCAGGCATCTTCACCAGG

D129G F: GAAGATGAGTGGCGGCCTGCTGGAGCTGG

R: CCAGCTCCAGCAGGCCGCCACTCATCTTC

S465L F: TGTGTGGTCAGCACACTTGGGGCCTACACGGATG

R: CATCCGTGTAGGCCCCAAGTGTGCTGACCACACA

P467A F: GGTCAGCACACTCGGGGGCTACACGGATGGCCAC

R: GTGGCCATCCGTGTAGCCCCCGAGTGTGCTGACC

I474V F: GATGGCCACAGCCGTCGCCCGCTGCG

R: CGCAGCGGGCGACGGCTGTGGCCATC

R499H F: AGCGGCGGGGCGAGCACATGGAGGCCCAAGG

R: CCTTGGGCCTCCATGTGCTCGCCCCGCCGCT

N513D F: GTCTGCCGGGCCCACGACGCTTTTGGGGGTG

R: CACCCCCAAAAGCGTCGTGGGCCCGGCAGAC

A514T F: TGCCGGGCCCACAACACTTTTGGGGGTGAGG

R: CCTCACCCCCAAAAGTGTTGTGGGCCCGGCA

G516V F: CACAACGCTTTTGTGGGTGAGGGTGTCTA

R: TAGACACCCTCACCCACAAAAGCGTTGTG
