# Supplementary figures and images for: Pathogenic gain-of-function mutations in the prodomain and C-terminal domain of PCSK9 inhibit LDL binding
Source: Front Physiol. 2022 Sep 14;13:960272. doi: 10.3389/fphys.2022.960272 (PMC9515655; doi:10.3389/fphys.2022.960272)

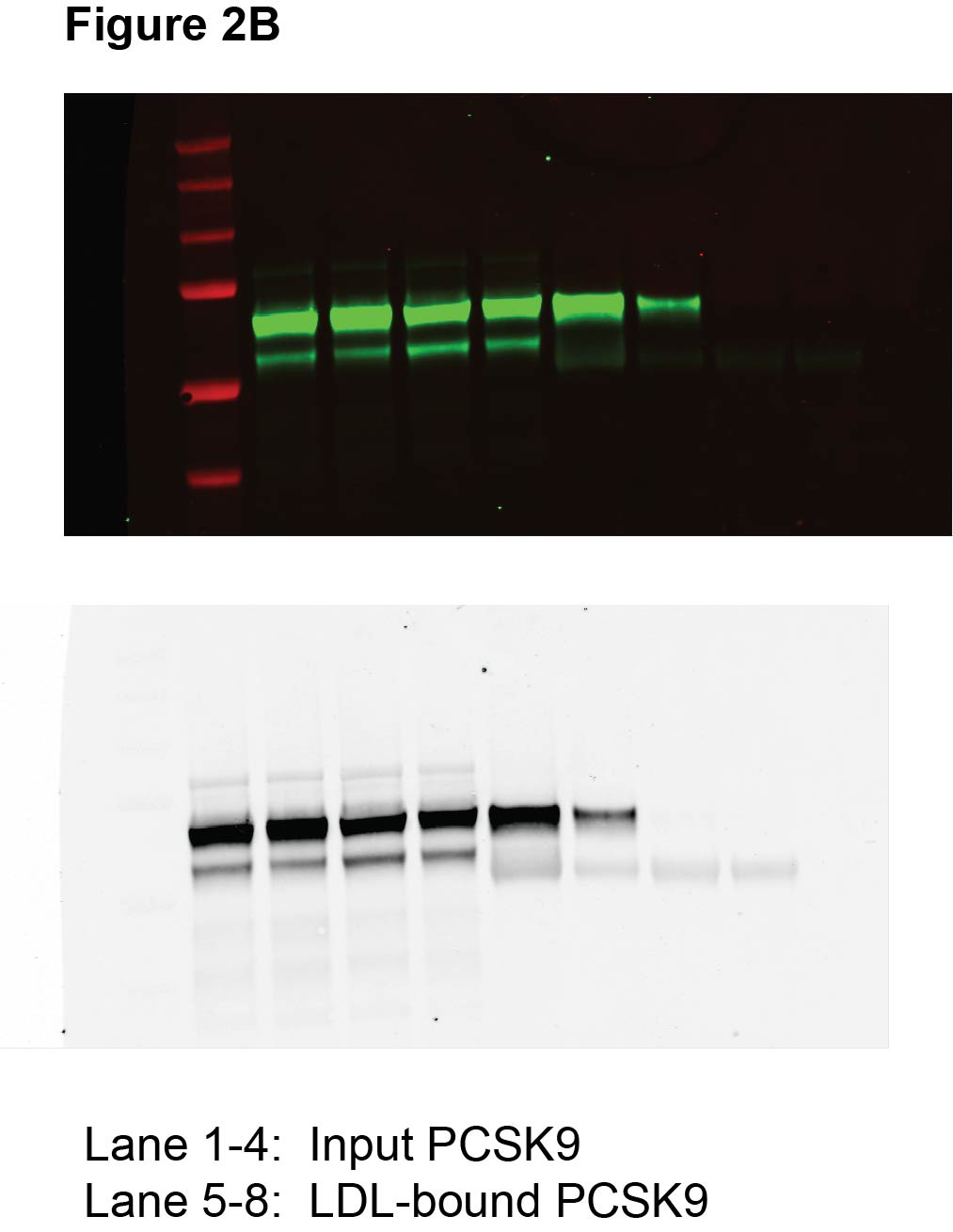


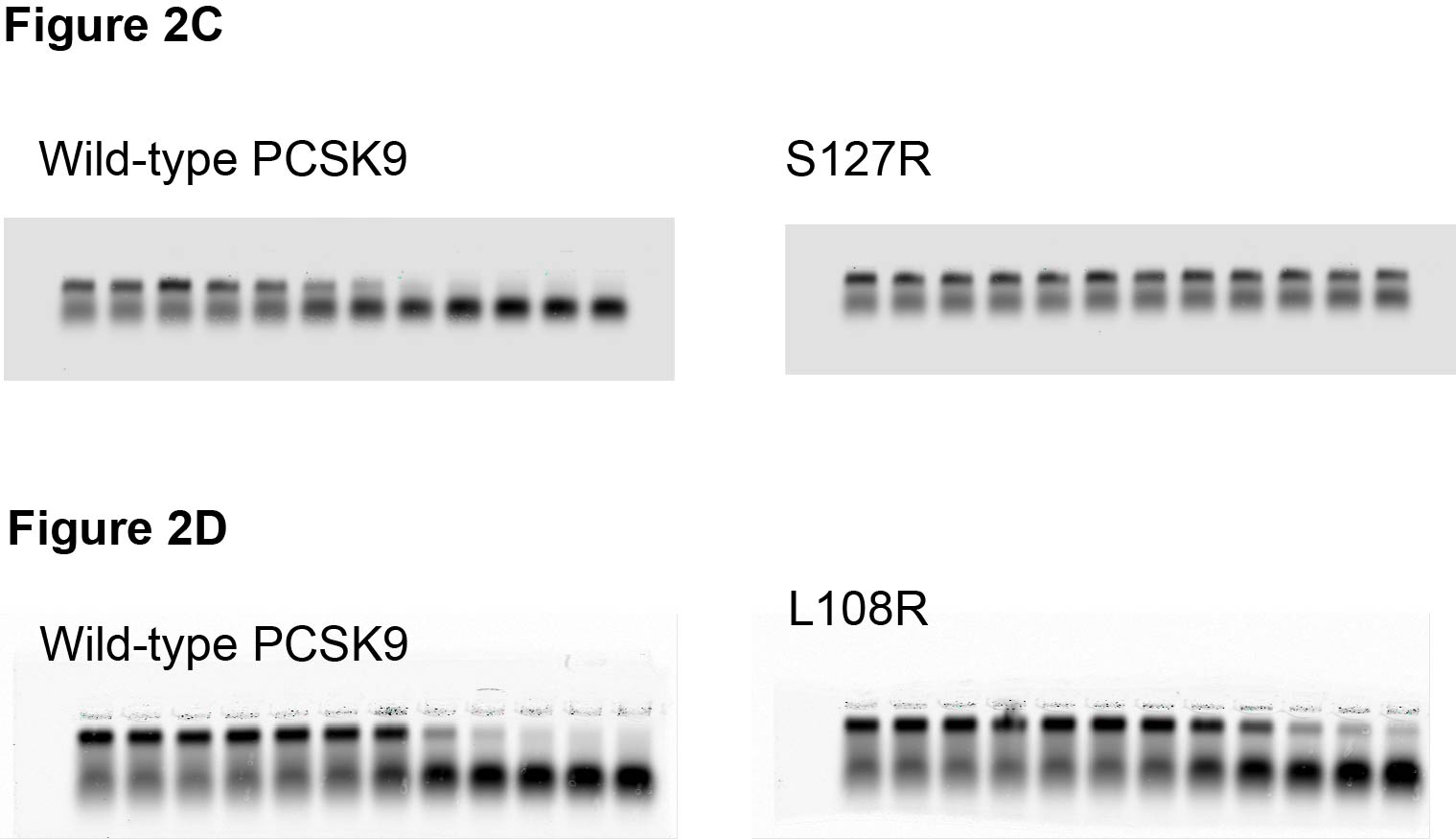


**Figure 3**

**
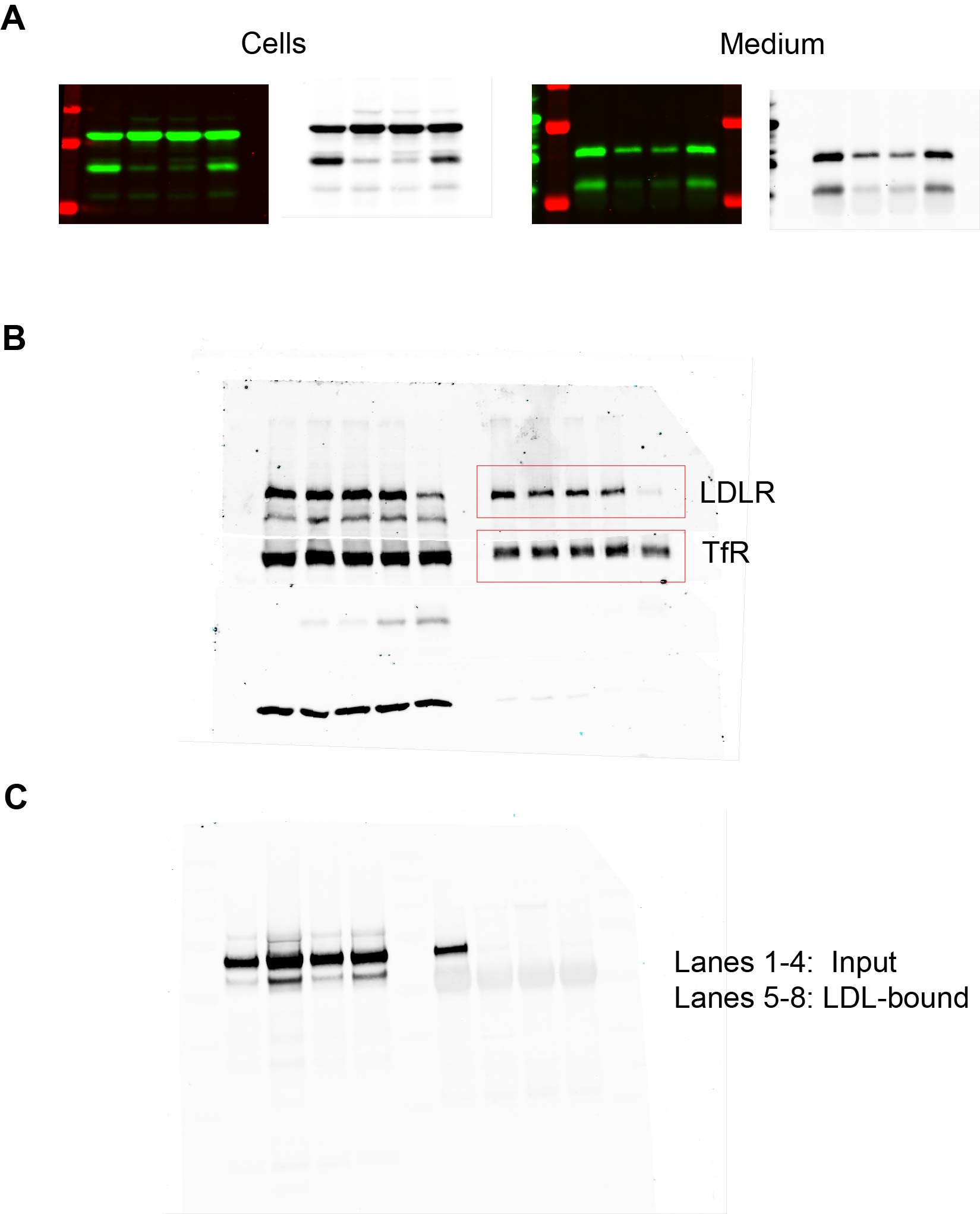
**

**Figure 4**

**
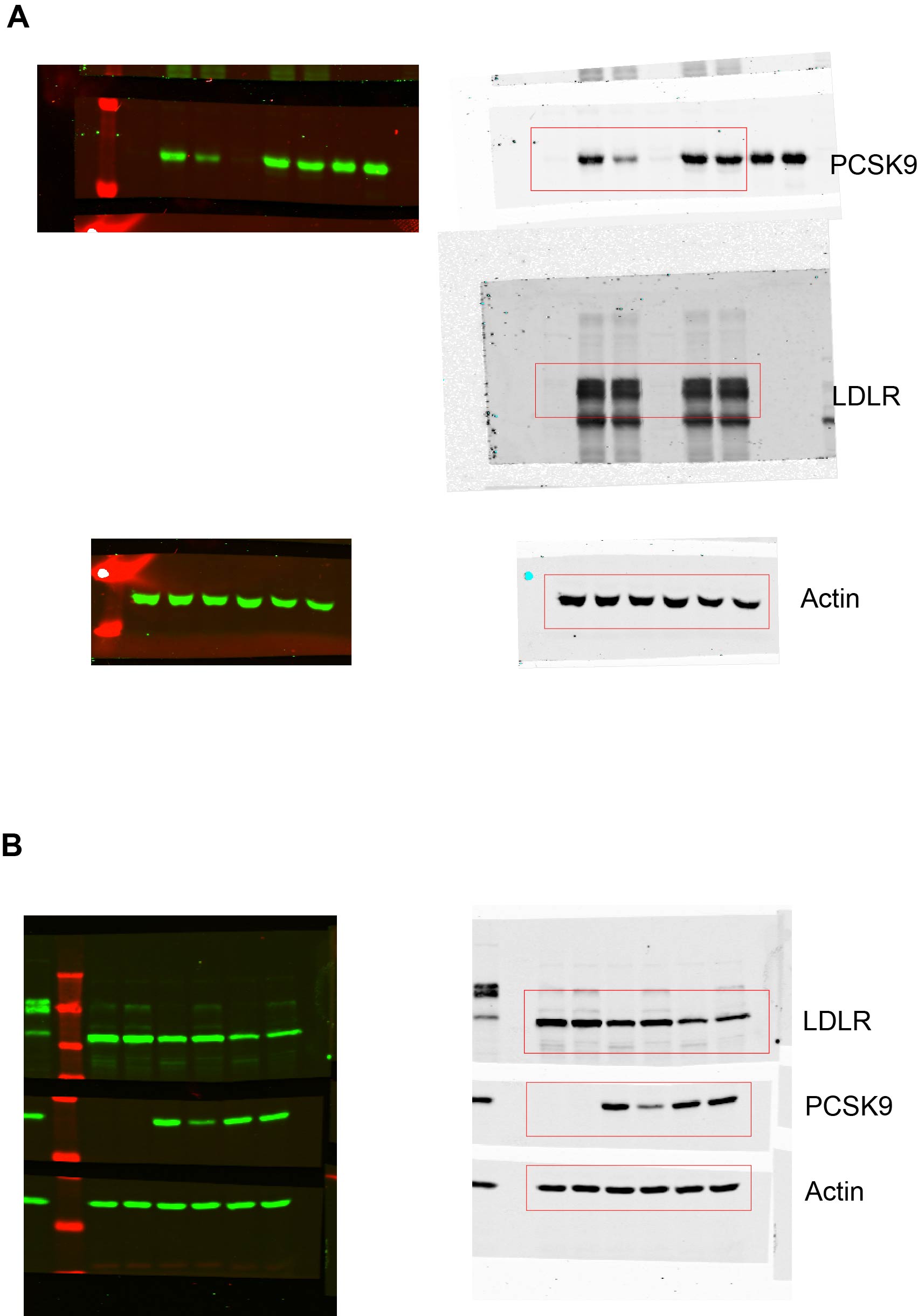
**

**Figure 5**

**
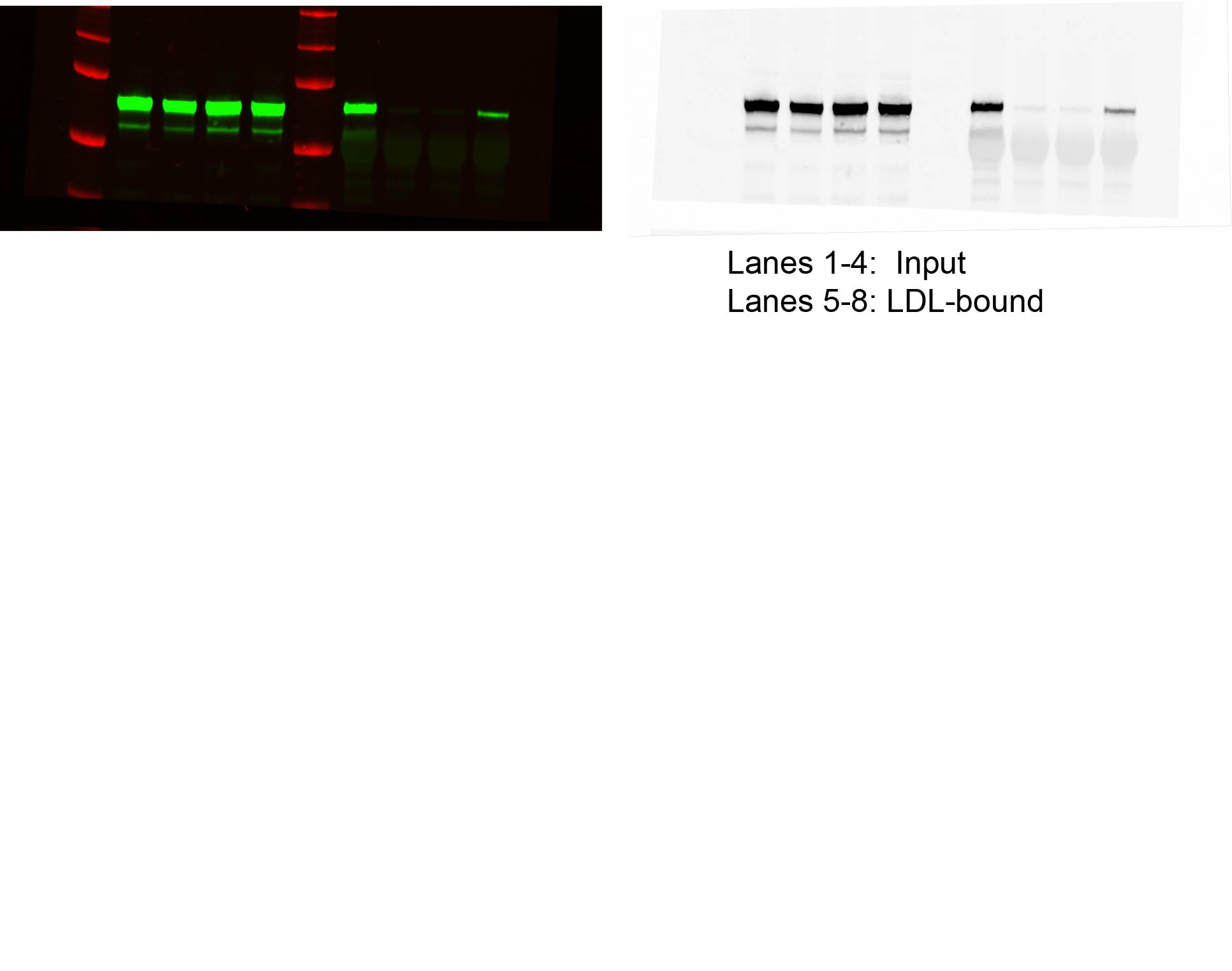
**

Supplement: Supplementary file 2 [file DataSheet1.DOCX]
